# Antioxidant properties of *Pelargonium graveolens L’Her* essential oil on the reproductive damage induced by deltamethrin in mice as compared to alpha-tocopherol

**DOI:** 10.1186/1476-511X-12-30

**Published:** 2013-03-07

**Authors:** Ahlem Ben Slima, Manel Ben Ali, Mohamed Barkallah, Al Ibrahim Traore, Tahia Boudawara, Noureddine Allouche, Radhouane Gdoura

**Affiliations:** 1 Unit Research of Toxicology-Microbiology Environmental and Health UR11ES70, Sciences Faculty of Sfax, University of Sfax, Sfax, Tunisia; 2 Laboratory of Chemistry of Natural Products, Sciences Faculty of Sfax, University of Sfax, B.P. 1171, Sfax, Tunisia; 3 National Laboratory of Public Health, Ouagadougou Burkina Faso, Burkina Faso, Cameroon; 4 Anatomopathology Laboratory, University of Sfax, CHU Habib Bourguiba, Sfax, 3029, Tunisia

**Keywords:** Deltamethrin, Oxidative damages, Geranium oil, Antioxidants

## Abstract

**Background:**

Exposure to the pyrethroid pesticide deltamethrin has been demonstrated to exert a wide range of effects on non-targeted organisms. The beneficial effects of geranuim essential oil (EO) as an antioxidant has been assessed in deltamethrin (DL) orally administered mice by studying whether the reprotoxicity caused by deltamethrin can be effectively combated with the geranium oil and the effects were compared to vitamin E, as the standard reference drug.

**Result:**

Sixty male albino mice were divided into six equal groups: a control group, a group of mice was given deltamethrin (5 mg/kg b.w.), two groups were administered deltamethrin after having given geranium essential oil (67 mg/kg b.w.) or vitamin E (Vit E) (100 mg/kg b.w.), and two groups received only EO of geranium or Vit E. When compared to control, a dose of deltamethrin 5 mg/kg/day causes a decrease in the epididymal sperm count motility and viability and an increase in the number of abnormal morphology in spermatozoa. DL-exposed mice showed a significant increase of lipid peroxidation (LPP) in the testis compared to control animals.

**Conclusion:**

Essential oil of geranium prevented testicular oxidative damage explored by reduced LPP and improved total sperm motility, viability and morphology in mice spermatozoa. Our study showed a positive influence of geranium essential oil in the animal male reproductive system similar than that of Vit E.

## Background

The increasing release of chemicals into the environment dictates attention to a better under-standing of their toxicity in human and ecotoxicological effects. Many studies suggest that environmental contaminants disrupt male reproduction and play an important role in the declination of the quality and quantity of human semen [1]. Several currently used pesticides are known to adversely impair reproductive competence of males under laboratory, field, clinical or occupational setting. Published studies reported that pyrethrinoid can impair fertility, deteriorate semen quality, and cause testicular degeneration, male reproductive failure and malformations in the foetuses in the rodents following repeated exposure [2,3]. Deltamethrin [(R, S)] is a type-II pyrethroid synthetic insecticide, which has been widely used to control noxious insects in agriculture, forestry and horticulture. However, a number of studies have demonstrated genotoxic and tumorogenic effects of deltamethrin in mammalian and non-mammalian species [4,5]. For many pesticides, induction of oxidative stress is one of the main mechanisms of their action. The chronic exposure to deltamethrin caused also haemolysis, hepatic, renal toxicities [6] and neurodegeneration [7]. The mechanism of such pathological facts may be prompted by the free radical release and the lipid peroxidation that it induces. During the past few years, estimation of free radical generation and antioxidants defense has become an important aspect of investigation in mammals for the protection of cells against oxidative damage due to pesticides [8], heavy metals [9] and chemotherapeutic agent toxicities that they generated probably oxygen-reactive species (ROS) which led to oxidative stress.

Rose-scented geranium (*Pelargonium graveolens L’Hér.*) is widely known as one of the medicinal herbs with the highest antioxidant activity. Essential oils are a folk medicine and recently their use has expanded worldwide to include therapy against various kinds of inflammatory diseases. A great number of new drugs discovered in the last few decades are originate from natural sources [10]. Natural products have been increasingly used for the prevention and treatment of various conditions [11,12].

*Geranium* essential oil has historically been used in the treatment of dysentery, hemorrhoids, inflammation, heavy menstrual flows and even cancer [13]. The French medicinal community currently treats diabetes, diarrhoea, gallbladder problems, gastric ulcers, liver problems, sterility and urinary stones with this oil [14,15]. In Chinese homeopathy, the geranium essential oil is known to open up the liver charka and promote the expulsion of toxins that prohibit the achievement of balance within the body [16]. The aim of the current study was to determine the components of *Pelargonium graveolens* essential oil and to evaluate its protective effects against deltamethrin-induced reprotoxicity in male mice.

## Material and methods

### Chemicals

DL is a synthetic pyrethroid insecticide (C_23_H_19_Br_23_NO_3_). CAS chemical name is a (1R,3R)-3-(2,2-dbromovinyl)-2,2-dimethyl-cyclopropane carboxylate de (S)-α-cyano-3 phénoxybenzyle, CAS registry number 52918-63-5. A commercial formulation of DL, named “Decis EC25” (Bayer CropScience) was used in the experiments. All other chemical products used in this study were purchased from Sigma Chemical Co. (St Louis, France).

### Plant material

Samples of *P.graveolens* (*geraniaceae*; known as Geranium); were collected from Sfax, South of Tunisia in March 2011. A voucher specimen was deposited at the laboratory of Natural substances chemistry, Faculty of Science of Sfax, Tunisia.

### Extraction of essential oil

A bout 200 g of the fresh leaves of *P.graveolens* was completely immersed in 1000 ml distilled water and hydro-distilled for 4 hours in Clevenger-type apparatus until 250 ml of the water–oil layer was obtained. The oily layer obtained on the top of the aqueous distillate was extracted three times with diethyl ether. The recovered organic solvent was dried over anhydrous sodium sulfate until the last traces of water were removed and evaporated by vacuum distillation at room temperature to yield greenish yellow oil. The obtained essential oil was stored in a dark glass bottle at 4°C until tested and analyzed. Yield based on dried weight of the sample was calculated.

### Gas chromatography–mass spectrometry (GC–MS)

The analysis of the essential oil was performed on a GC–MS HP model 5975B inert MSD (Agilent Technologies, J&W Scientific Products, Palo Alto, CA, USA), equipped with an Agilent Technologies capillary DB-5MS column (30 m length; 0.25 mm i.d.; 0.25 μm film thickness), and coupled to a mass selective detector (MSD5975B, ionization voltage 70 eV; all Agilent, Santa Clara, CA). The carrier gas was He and was used at 1 ml/min flow rate. The oven temperature program was as follows: 1 min at 100°C ramped from 100 to 260°C at 4°C min-1 and 10 min at 260°C. The chromatograph was equipped with a split/splitless injector used in the split mode. The split ratio was 1:100. Identification of components was assigned by matching their mass spectra with Wiley and NIST library data, standards of the main components and comparing their Kovats Retention Indices (KRI) with reference libraries and from the literature [17,18]. The component concentration was obtained using semi-quantification by peak area integration from GC peaks and by applying the correction factors.

### Animals care

Adult, healthy and virgin Swiss Albino male mice weighing 27 ± 3.0 g were obtained from the Centre of Veterinary Research (Sfax, Tunisia). Animals were examined for health status and acclimated to the laboratory environment for 1 week *prior* to use. All animals were housed in stainless steel cages and maintained on a 12 h light /dark cycle. The animal room was designed to maintain temperature at 23 ± 2°C and relative humidity at approximately 50%. Food and water were provided *ad libitum*.

### Experimental design

The animals were randomly divided into six groups of ten mice each and they were treated as follows: group 1: control mice received 2.5% diluted DMSO by gavages (C), and served as negative control; group 2: received DL (5 mg/kg b.w./day (≈ ¼ DL50 for mouse)); group 3: received DL(5 mg/kg b.w./day) and essential oil of *P.graveolens* at (67 mg/kg b.w./day) after 120 min of DL administration; group 4: received DL(5 mg/kg body weight/day) and Vitamin E (Vit E) at 100 mg/kg b.w./day after 120 min of DL administration; group 5: received only essential oil *of P.graveolens* (EO) at 67 mg/kg b.w./day; group 6: received only Vitamin E (Vit E) at dose 100 mg/kg body weight, and served as positive control for antioxidant treatment. The essential oil and Vitamin E were diluted to 2.5% by 2.5% dimethyl sulfoxide (DMSO) and then we added 12.5 μl/ml of Tween20 [19].

The experimental was continued for 35 days. The proper doses of treatment for each animal were placed into a syringe that was inserted orally with the help of plastic tube directly into the oropharyngeal region. The experimental protocol was performed according to the European convention for the protection of vertebrate animals used for experimental and other scientific purposes (Council of Europe No123, Strasbourg, 1985) and approved by the ethics committee for research on laboratory animal use of our institution.

### Sperm quality

Epididymal sperm were collected by cutting the one epididymis into small pieces in 2 ml of physiological saline at 32°C. Sperm evaluation included cell concentration, viability, progressive motility, and normal morphology according to World Health Organization guidelines [20].

#### Sperm motility and count

Sperm motility was determined by scoring the number of all progressive sperm and then the non-progressive and the immotile sperm in the same field. On each slide, at least 100 sperm cells were counted. Sperm motility assessment was repeated in a new preparation of the same sample. First, the percentage of sperm cells in each motility group was calculated and then the average in each group for the two assessments of the two slides was assayed according to Kvist and Björndahl [21]. From this solution, 20 μl aliquots were placed on the Neubauer Hemacytometer for counting the number of spermatozoa/epididymis.

#### Sperm viability

Sperm viability study was assessed using the Eosin/Nigrosin stain. The staining was performed with 1 drop of fresh semen into 2 drops of staining solution on a microscope slide. Using another slide, a smear was made and allowed to dry. Unstained (intact) and red-colored (with damaged membranes) spermatozoa were counted under the microscope using × 1000 objectives and oil immersion. Sperm viability was defined as the percentage of intact cells, as per the procedures described in the WHO Laboratory Manual [22].

#### Sperm morphology

To evaluate the spermatozoa abnormalities, sperm suspension was stained with Eosin; smears were made on slides, air-dried and made permanent. The spermatozoa were classified according to the Wyrobek and Bruce criteria [23]. Sperm cells with normal morphology and cells presenting abnormalities in head, mid-piece and tail were assessed. At least 200 sperm cells were observed in each slide, under 1000× magnification.

### Biochemical assays

#### Measurement of Lipid Peroxidation and Protein Carbonyl Levels in testis

LPP process is determined by the thiobarbituric acid (TBA) method which estimates the malondialdehyde formation (MDA) [24]. Briefly, 0.5 ml of testis homogenate was mixed with 1 ml of trichloroacetic acid solution and centrifuged at 2500 g for 10 min. One millilitre of a solution containing 0.67% thiobarbituric acid (TBA) and 0.5 ml of supernatant were incubated for 15 min at 90°C and cooled. Absorbance of TBA-MDA complex was measured at 532 nm. Lipid peroxidation is expressed as nmoles MDA/g tissue.

Protein oxidation was determined based on the reaction of the carbonyl groups with 2, 4-dinitrophenylhydrazine (DNPH) to form 2, 4 dinitrophenylhydrazone, as described by Reznick and Packer [25]. Samples were read at 370 nm and carbonyl content was calculated using the molar absorption coefficient for aliphatic hydrazones (22,000 M^-1^ cm^-1^) and expressed as μmole carbonyl/mg protein.

#### Determination of testis enzymatic antioxidants

Catalase (CAT) was assayed by the decomposition of hydrogen peroxide and converts it to water and molecular oxygen according to the method of Aebi [26]. Decrease in absorbance due to H_2_O_2_ degradations was monitored at 240 nm for 1 min and the enzyme activity was expressed as μmol H_2_O_2_ consumed /min/mg protein.

Superoxide dismutase (SOD) activity was estimated according to Beauchamp and Fridovich [27]. The reaction mixture contained 50 mM of testis tissue homogenates in potassium phosphate buffer (pH = 7.4), 0.1 mM methionine, 2 μM riboflavine and 75 μM Nitro Bleu Tetrazoluim (NBT). The developed blue colour reaction was measured at 560 nm. Units of SOD activity were expressed as the amount of enzyme required to inhibit the reduction of NBT by 50% and the activity was expressed as U/ mg protein.

#### Determination of testis non-enzymatic antioxidants

GSH in tissues was determined by the method of Ellman [28] modified by Jollow et al [29] based on the development of a yellow colour when 5, 5-dithiobis-2nitrobenzoicacid (DTNB) was added to compounds containing sulfhydryl groups. 500 μl of testis tissue homogenate in phosphate buffer were added to ml of 4% sulfosalicylic acid. The mixture was centrifuged at 1600 g for 15 min. 500 ml of supernatant were taken and added to Ellman’s reagent. The absorbance was measured at 412 nm after 10 min. total GSH content was expressed as μg/mg of tissue.

Ascorbic acid content was determined spectrophotometrically by dinitrophenyl-hydrazine method described by Jacques-Silva and others [30]. Briefly, the ascorbic acid in the testes homogenate was oxidized by Cu ^2+^ to form dihydro-ascorbic acid, which reacts with acidic 4-dinitrophenylhydrazine to form a red hydrazone. Final color development was achieved with 65% sulfuric acid and then optical density was measured at 540 nm. The concentration of the samples in vitamin c is obtained from a standard range of ascorbic acid from a dilution of 10 mg/ml ascorbic acid.The calibration curve was prepared using ascorbic acid as standard. Results were expressed as μmole/g of tissue.

#### Protein determination in testis

The total protein concentration of supernatant was determined according to Lowry et al [31] using bovine serum albumin BSA as a standard.

### Histopathology

For light microscopic examination, tissue samples of testis of the experimental mice were fixed in 4% paraformaldehyde solution for 24 h. After routine processing, paraffin-embedded tissue samples were sectioned at 3-5 μm thickness and stained with Mayer’s haematoxylin and eosin. For testicular toxicity, approximately 200 circularly sectioned seminiferous tubules for each mouse were assessed microscopically, and then the percentages of tubules with histopathological changes were calculated [32].

### Statistical analyses

The results obtained are expressed as mean values ± SD. Statistical significance was assessed using one-way analysis of variance (ANOVA). Values of P < 0.05 were regarded as statistically significant. All statistical analyses were performed using the Graph Pad Prism version 4 Software (Graph Pad Software, Inc. La Jolla, CA, USA).

## Results and discussion

The result obtained with GC/MS analysis of the oil is depicted in Table 1. The essential oil was found to contain 18 constituents representing 89.04% of the total essential oil while minor constituents (10.96%) of the oil remained unidentified. The most abundant components (> 4%) of the *P. graveolens* essential oil were β-citronellol (29.3%) followed by geraniol (10.53%), linalool (10.42%) and citronellal formate (9.54%). These results were in agreement with previous studies [33,34] which demonstrated that the geranium leaves essential oil contains high levels of these compounds. Further, Bouaziz and his collaborators were proved that the antioxidant activity of the essential oil could be attributed in part to the presence of compounds such as β -citronellol and geraniol and its ability to decompose free radicals by quenching reactive oxygen species and trapping radicals before reaching their cellular targets [35].

**Table 1:** **Chemical composition of *P. graveolens *essential oil**

**N°**	**KI**^**a**^	**Compound**	**Composition (%)**	**TR**^**b**^
1	939	α-pinene	0.54	8.67
2	1031	β-phellandrene	0.10	11.55
3	1040	cis-ocimene	0.08	12.03
4	1098	linalool l	10.42	13.82
5	1127	rose oxide-trans	0.6	14.62
6	1154	menthone	7.34	15.22
7	1189	α-terpineol	2.87	16.05
8	1233	β-citronellol	29.30	17.5
9	1255	geraniol	10.53	18.33
10	1275	citronellyl formate	9.54	18.81
11	1290	Thymol	7.84	19.02
12	1418	Trans-caryophyllene	1.2	20.5
13	1439	aromadendrene	0.91	23.56
14	1480	germacrene-d	3.46	29.98
15	1513	γ-cadinene	1.20	24.91
16	1514	leden	1.63	25.08
17	1524	δ-cadinene	1.25	25.33
18	1530	epizonaren	0.21	25.42
**Identified components (%)**		**89.04**%		
**Monoterpenes hydrocarbons**		**0.72**		
**Oxygenated monoterpenes**		**68.9**		
**Sesquiterpenes hydrocarbons**		**9.88**		
**Oxygenated sesquiterpenes**		**-**		
**Other compounds**		**9.54**		

TR^b^ : Retension Time (min) KI^a^ : Retention Index (kovats).

**Figure 1:**
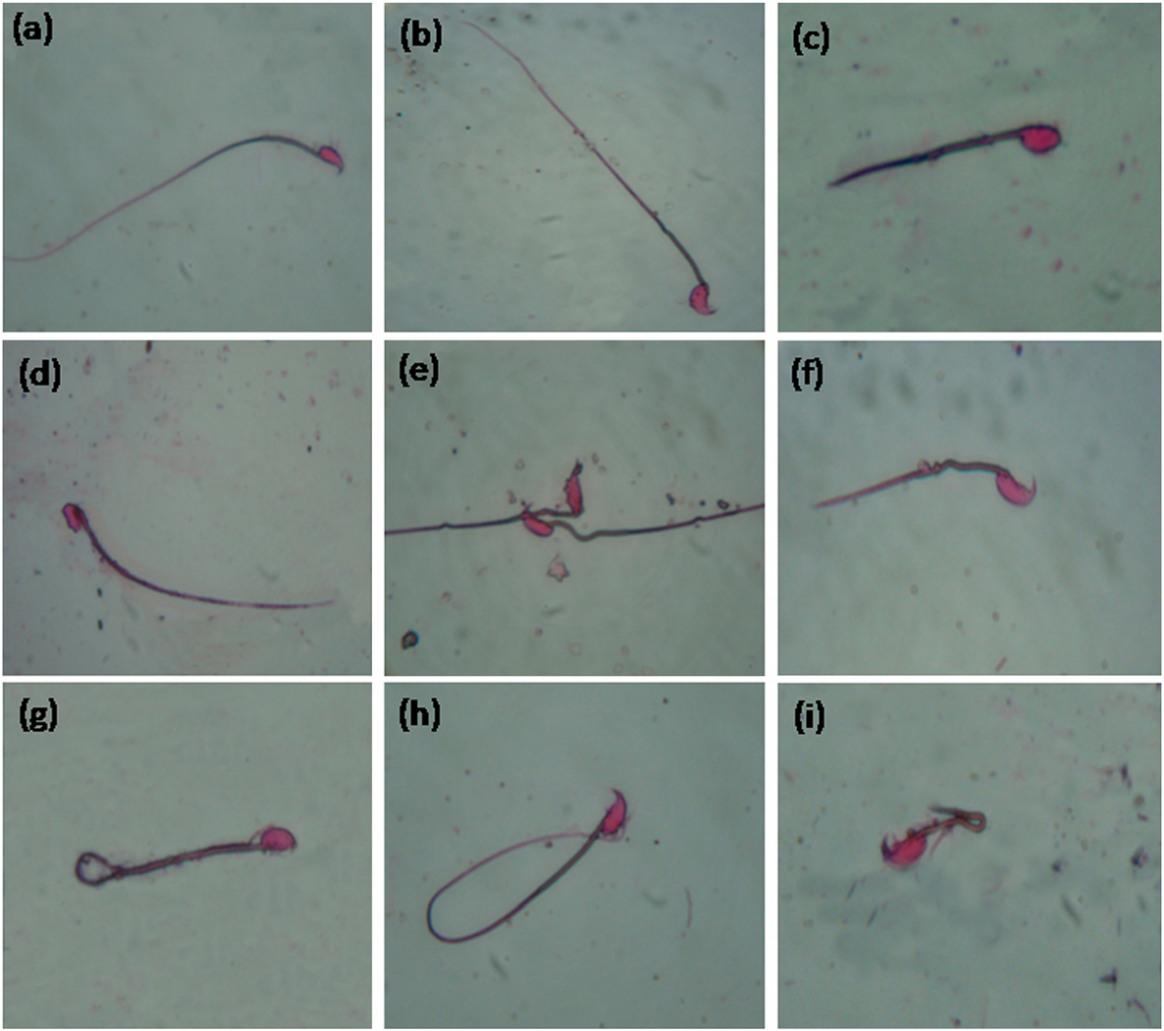
Eosin-stained spermatozoa of mice, (a-b) Control; (c-d) head abnormalities of the spermatozoa; (e-f) mid-piece abnormalities and (g-i) tail abnormalities.

Xenobiotics and environmental contaminants such as pesticides are known to induce a broad spectrum of toxicological effects and biochemical dysfunctions constituting serious hazards to health. The epididymal sperm count motility and viability were significantly decreased (p ≤ 0.001) after DL administration (Table 2) whereas a significant increase was observed in abnormal morphology of spermatozoa (head anomalies (Figure 1c-d); mid-piece anomalies (Figure 1e-f) and tail anomalies (Figure 1g-i)) compared to the control group (Figure a-b). These anomalies are counted in each group and classified according to their intensities in Table 3. Thus, our results support and extend previous reports suggesting that synthetic pyrethroid generally impairs sperm characteristics [36].

**Table 2:** **Effect of different treatments on cell’s density, viability, motility and morphology in control group (C) and mice treated with deltamethrin (DL), essential oil of *P. graveolens *(EO) and Vitamin E (Vit E) or their combination**

	**Control**	**DL**	**DL + EO**	**DL + Vit E**	**EO**	**Vit E**
**Spermatozoa count** Per epididymis (×10^6^)	5.90 ± 0.55	1.96 ± 0.25***	5.09 ± 0.35*^###^	5.11 ± 0.31*^###^	5.77 ± 0.62^**###**^	5.76 ± 0.71^**###**^
**Sperm parameters** Motility **(%)**	78.44 ± 5.85	39.33 ± 6.82***	76.11 ± 10.01^**###**^	66.11 ± 18.6^**###**^	75.00 ± 8.87^**###**^	88.67 ± 2.74^**###**^
Viability **(%)**	89.33 ± 3.97	72.11 ± 3.14***	83.22 ± 1.92*^###^	83.00 ± 4.61*^###^	85.89 ± 6.13^**###**^	89.00 ± 4.27^**###**^
Morphology **(%)**	7.00 ± 1.58	22.00 ± 3.94***	15.53 ± 3.59**^#^	16.88 ± 7.12**^#^	6.25 ± 3.28^**###**^	7.88 ± 3.00^**###**^

Data are presented as mean ± SD. All groups vs. control group: * P < 0.05; ** P < 0.01; *** P < 0.001. All groups vs. DL group: # P < 0.05; ## P < 0.01; ### P <0.001.

**Table 3:** **Results of sperm morphology assessment in control group (C) and mices treated with deltamethrin (DL), essential oil of *P. graveolens *(EO) and Vitamin E (Vit E) or their combination**

		**Control**	**DL**	**DL + EO**	**DL + Vit E**	**EO**	**Vit E**
**Categories of sperm abnormality in semen samples**	**Head-abnormalities**	**-**	**++++**	**+**	**+**	**-**	**-**
	**Mid-piece abnormalities**	**-**	**++++**	**-**	**-**	**-**	**-**
	**Tail abnormalities**	**+**	**++++**	**+**	**+**	**+**	**+**

+++ Severe intensity of the anomaly, + moderate to low intensity of the anomaly, - very low intensity of the anomaly.

**Figure 2:**
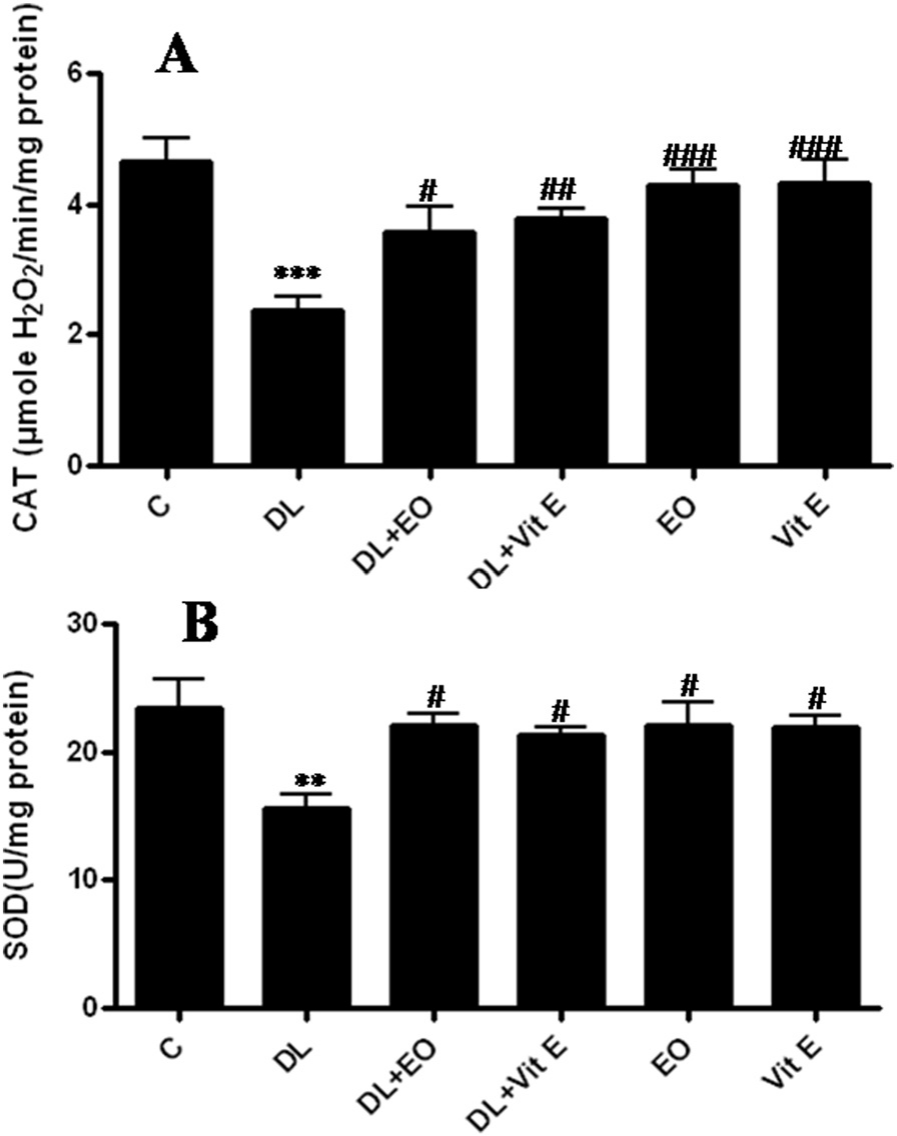
**Effect of different treatments on CAT activity (part A) and SOD activity (part B) in testis of controls (C) and mice treated with deltamethrin (DL), essential oil of *P.graveolens *(EO),Vitamin E (Vit E) or their combination.** Values are expressed as means ± SD of ten mice in each group. All groups vs. control group: ** P < 0.01; *** P < 0.001. All groups vs. DL group: ^#^ P < 0.05; ^##^ P < 0.01; ^###^ P < 0.001.

**Figure 3:**
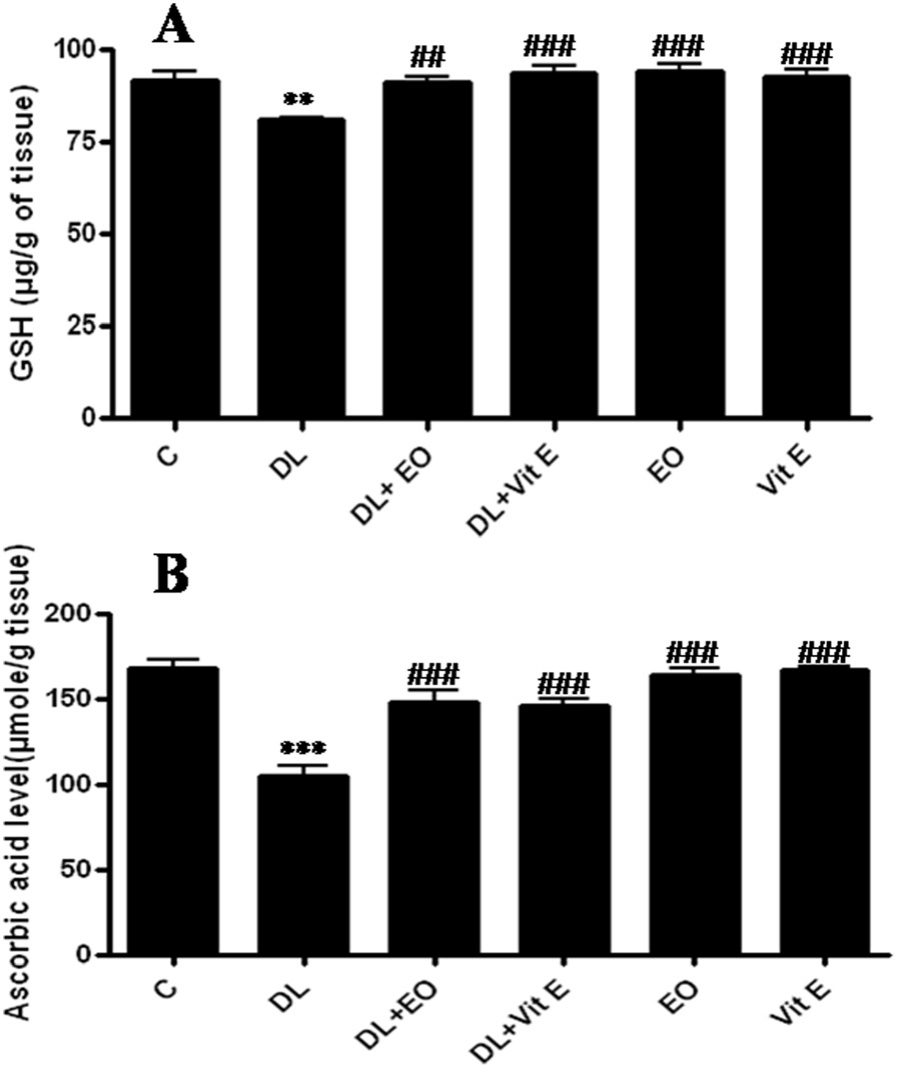
**Effect of different treatments on GSH activity (part A) and Ascorbic Acid levels (part B) in testis of controls (C) and mice treated with deltamethrin (DL), essential oil of *P.graveolens *(EO),Vitamin E (Vit E) or their combination.** Values are expressed as means ± SD of ten mice in each group. All groups vs. control group: ** P < 0.01; *** P < 0.001. All groups vs. DL group: ^##^ P < 0.01; ^###^ P < 0.001.

**Figure 4:**
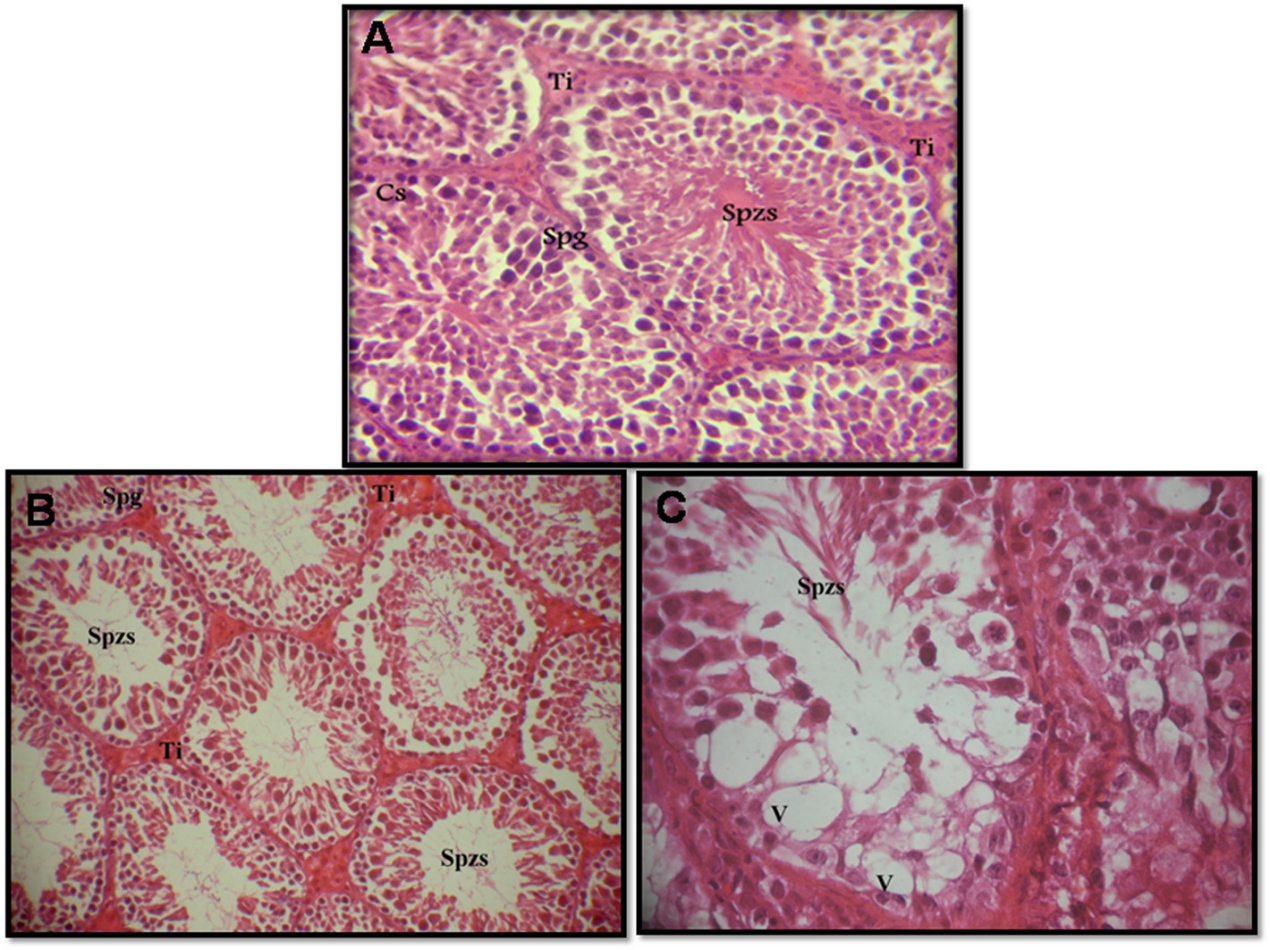
**Testicular sections of control mice which show normal spermatogenesis (part A): Note the normal cell arrangement in the seminiferous tubules.** The interstitial spaces also appear normal Ti, interstitium; Sg, spermatogonia; Sd, spermatid; Spzs, spermatozoa; CS, sertoli cell (x 200 H&E). Testicular sections of mice treated with 5 mg/kg/day of DL: Note the atrophic seminiferous tubules (part **B**) (x400 H&E). Sloughing of germ cells into tubular lumen, V, vacuolization in Sertoli cells and (arrow) multinucleated giant cell (part **C**) (x1000 H&E).

**Figure 5:**
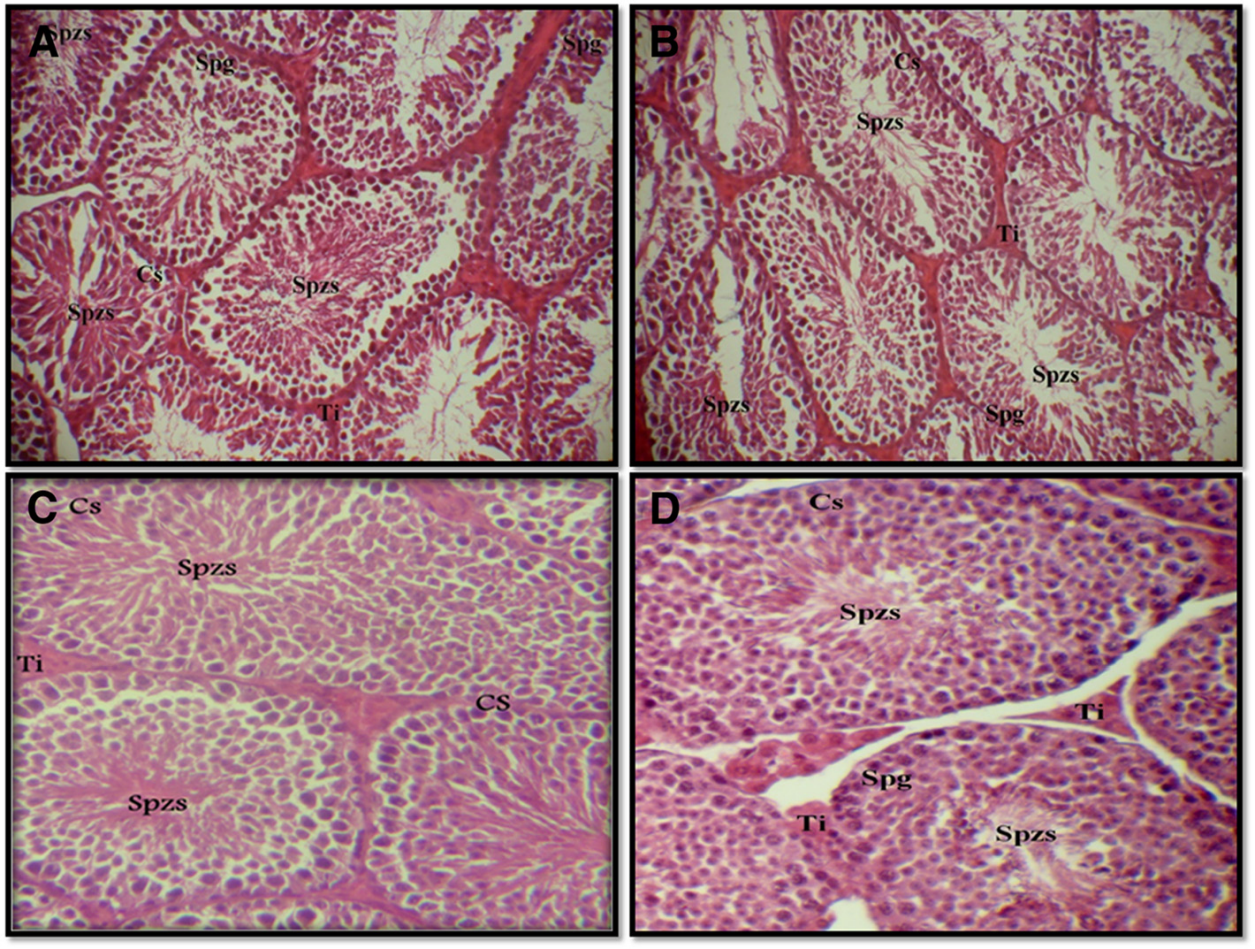
**Testicular sections of mice treated with 5 mg/kg/day of DL and 100 mg/kg Vit E (figure A) or DL + EO (part B): Note the increase of germ cells in the seminiferous tubules.** The interstitial spaces also appear normal Ti, interstitium; Sg, spermatogonia; Sd, spermatid; Spzs, spermatozoa; CS, sertoli cell (x 400 H&E). Testicular sections of mice treated with Vit E (part **C**) or EO (part **D**): Note the normal cell arrangement in the seminiferous tubules. The interstitial spaces also appear normal Ti, interstitium; Sg, spermatogonia; Sd, spermatid; Spzs, spermatozoa; CS, sertoli cell (x 400 H&E).

The reduction in sperm count may be due to an adverse effect of DL on spermatogenesis. Lipid peroxidation (LPP) is a marker of oxidative damage. DL-exposed mice showed a significant increase (p ≤ 0.01) of LPP and protein carbonyls content in the testis compared to control animals (Table 4). Increased lipid peroxidation and altered membrane function can render sperm dysfunctional through impaired metabolism, motility, acrosome reaction reactivity and fusogenic capacity as well as oxidative damage to sperm DNA [37,38]. These results were similar to those of Samah et al [39] who reported the DL induced LPP production in testis rats. The DL induced reduction in testicular CAT activity (Figure 2) may reflect less capacity of testicular mitochondria and microsomes to eliminate H_2_O_2._ SOD is considered the first line of defense against deleterious effects of free radicals in the cell by catalyzing the dismutation of superoxide radicals to hydrogen peroxide and molecular oxygen. Testicular SOD activity (Figure 3) declined significantly (p ≤ 0.01) in the testis of DL-treated animals when compared with control animals. Thus, our results support and extend previous reports suggesting that pesticide intoxication generally impairs the testicular antioxidant defense system and induces lipid peroxidation in experimental animals and humans [40,41]. Wang et al [42] have also reported that Beta-Cypermethrin decreased the activity of both CAT and SOD. Yet, these activities increased after antioxidants supplementation. We have also investigated the contribution of GSH and vitamin C against oxidative stress. DL treatment led to a significant decrease in GSH (P < 0.01) (Figure 4) and ascorbic acid (P < 0.001) levels in testis (Figure 5) compared with control group. GSH is an important naturally occurring antioxidant, which prevents free radical damage and helps detoxification by conjugating with chemicals. Under oxidative stress, GSH is consumed by GSH related enzymes to detoxify the peroxides produced due to increased lipid peroxidation [43]. These effects were significantly mitigated by 67 mg/kg EO co-treatment compared with DL-treated group. However, in essential oil supplemented groups the LPP and protein carbonyls declined significantly with relative to DL-exposure group. Our results showed that deltamethrin induced oxidative stress in the mice testis may be successfully treated with the essential oil, geranium, through its antioxidant effects. In this setting, geranium prevented testicular oxidative damage, reduced lipid peroxidation and improved total sperm motility, viability and morphology in mice spermatozoa. The histological results reported in the current study confirmed the biochemical results. In light microscopic examinations, DL-exposed mice showed severe histopathological changes in the testis such as atrophic seminiferous tubules (Figure 4B). The affected tubules were lined by fewer spermatogenic cells. In addition, appearance of multinucleated giant cells, vacuolization in Sertoli cells and loss of germ cells were also found (Figure 4C). However, the essential oil and vitamin E administration to DL group reduced the appearance of histo-pathological changes in testis (Figure 5 A-D). The testes of control group (Figure 4A) and animals administrated with EO and Vit E (Figure 5 C-D respectively) showed a normal histological structure. In fact, seminiferous tubules presented a complete spermatogenesis and an increase in spermatozoa inside the lumen of seminiferous tubules. Similar histological changes in the testis and epidydimis have been documented previously [39]. The administration of essential oil of *P.graveolens* exerts a strong protective effect against oxidative stress damage at the protein level. The essential oil at a dose of 67 mg/kg/day was effective in the reduction of proteins oxidation in testis induced by deltamethrin toxicity. The reduced oxidative stress and lipid peroxidation observed in the geranium essential oils treated animals may be attributed to the important role of the essential oil as antioxidants. The geraniol and β-citronellol may have influenced the geranium essential oil effects as they were major components of the oil (Table 1). In addition, geraniol has been found to have significant antioxidant properties that might be effective for many problems and stabilized and protected the cell membrane against oxidative stress [44]. Numerous studies (in animals and infertile humans) reported a beneficial effect of vitamin E supplementation on sperm quality: reducing the amount of TBARS (marker of lipid peroxidation) in seminal plasma with improved mobility and velocity of sperm, increase concentration and decrease abnormal sperm [45]. Administration of EO at dose of 67 mg/kg bw, 2 hours after DL exposure showed the same effect as vitamin E and has improved the poor quality of sperm induced by deltamethrin which cause higher motility score in the spermatozoa. Their positive effects stem from their ability to inhibit lipid peroxidation, chelate redox- active metals, and attenuating other processes involving reactive oxygen species [46,47].

**Table 4:** **Effect of different treatments on oxidative stress parameters: lipid peroxidation (MDA) and protein carbonyl content (PCO) in testis of controls (C) and mice treated with deltamethrin (DL), essential oil of *P. graveolens *(EO) and Vitamin E (Vit E) or their combination**

	**C**	**DL**	**DL + EO**	**DL + Vit E**	**EO**	**Vit E**
**MDA**^a^	299.26 ± 11.64	470.93 ± 11.02***	303.8 ± 13.88^**###**^	297.46 ± 5.57^**###**^	242.20 ± 8.72^**###**^	284.53 ± 12.10^**###**^
**PCO**^**b**^	0.30 ± 0.10	0.92 ± 0.25***	0.46 ± 0.10^**##**^	0.47 ± 0.08^**##**^	0.23 ± 0.13^**###**^	0.53 ± 0.08^**###**^

^a^ (nmoles/g); ^a^ (μmoles carbonyl/mg protein). Values are expressed as means ± SD of ten mice in each group. All groups vs. control group: * P < 0.05; ** P < 0.01; *** P < 0.001. All groups vs. DL group: # P < 0.05; ## P < 0.01; ### P <0.001.

To the best of our knowledge, this study is the first *in vivo* experimental that investigated the antioxidant effect of *Pelargonium graveolens* on mice sperm kinematic parameters.

The rose-scented geranium *Pelargonium graveolens L’Her* is used for many years in traditional medicine [48,49]. Recently, Boukhris et al demonstrated that administration of essential oil of *P.graveolens* may be helpful in the prevention of diabetic complications associated with oxidative stress [50]. It seems that many of these pharmacologic features of essential oil of geranium are due to its potent antioxidant actions. In the present study, geranium acts as a natural suppressor of oxidative stress which resulting in sperm promoted quality.

## Conclusion

Based on these results, this study showed a positive influence of geranium essential oil in the animal male reproductive system. Further preclinical studies and clinical trials in humans are needed to find out a possible place for it in therapies of fertility disturbances.

## Competing interests

The authors declare that they have no competing interests.

## Authors’ contributions

AB; MB and MB designed the experiments, analyzed the data and drafted the manuscript. TB, helped in histopathology. AT helped in data analysis. NA and RG conceived research and approaches and have given final approval of the manuscript to be published. All authors read and approve the final manuscript.

## References

[B1] Figa-Talamanca I Traina ME Urbani E London: Occupational exposures to metals, solvents and pesticides: recent evidence on male reproductive effects and biological markers Occup Med 2001 51 174 188 10.1093/occmed/51.3.174 11385122

[B2] Wang H Wang SF Ning H Ji YL Zhang C Zhang Y Yu T Ma XH Zhao XF Wang Q Liu P Meng XH Xu DX Maternal cypermethrin exposure during lactation impairs testicular development and spermatogenesis in male mouse offspring Environ Toxicol 2011 26 4 382 394 10.1002/tox.20566 20131380

[B3] Weselak M Arbuckle TE Wigle DT Walker MC Krewski D Pre- and post-conception pesticide exposure and the risk of birth defects in an Ontario farm population Reprod Toxicol 2008 25 472 480 10.1016/j.reprotox.2008.05.060 18586452

[B4] Husain R Adhami VM Selth PK Behavioral, neurochemical, and neuromorphological effects of deltamethrin in adult rats J Toxicol Environ Health 1996 48 515 526 10.1080/009841096161212 8751838

[B5] Ismail MF Mohamed HM Deltamethrin-induced genotoxicity and testicular injury in rats: comparison with biopesticide Food Chem Toxicol 2012 50 3421 3425 10.1016/j.fct.2012.07.060 22889898

[B6] Saoudi M Messarah M Boumendjel A Jamoussi K El Feki A Protective effects of Vitamin C against haematological and biochemical toxicity induced by deltamethrin in male Wistar rats Ecotoxicol Environ Saf 2011 74 1765 1769 10.1016/j.ecoenv.2011.04.003 21514672

[B7] Hossain MM Richardson JR Mechanism of pyrethroid pesticide–induced apoptosis: role of Calpain and the ER stress pathway Toxicol Sci 2011 122 2 512 525 10.1093/toxsci/kfr111 21555338 PMC3155085

[B8] Yousef MI Awad TTI Mohamed EH Deltamethrin-induced oxidative damage and biochemical alterations in rat and its attenuation by Vitamin E Toxicology 2006 227 240 247 10.1016/j.tox.2006.08.008 16978760

[B9] Prasenjit M Mahua S Sil PC Cadmium induced testicular pathophysiology: pophylactic role of Taurine Reprod Toxicol 2008 26 282 291 10.1016/j.reprotox.2008.09.009 18926901

[B10] Newman DJ Cragg GM Natural products as sources of new drugs over the last 25 years J Nat Prod 2007 70 477 10.1021/np068054v 17309302

[B11] Thomas KJ Nicholl JP Coleman P Complement Ther Med 2001 9 2 10.1054/ctim.2000.0407 11264963

[B12] Mainardi T Kapoor S Bielory L Complementary and alternative medicine: herbs, phytochemicals and vitamins and their immunologic effects J Allergy Clin Immunol 2009 123 294 10.1016/j.jaci.2008.12.023 19203652

[B13] Kang HY Na SS Kim YK Effects of oral care with essential oil on improvement in oral health status of hospice patients J Korean Acad Nurs 2010 40 4 473 481 10.4040/jkan.2010.40.4.473 20820114

[B14] Amabeoku GJ M Antidiarrhoeal activity of Geranium incanum Burm of (Geraniaceae) leaf aqueous extract in mice J Ethnopharmacol 2009 123 Issue 1, 4 190 193 19429361 10.1016/j.jep.2009.02.015

[B15] Elmann A Mordechay S Rindner M Ravid U Anti- neuroinflammatory effects of geranium oil in microglial cells Journal of Functional Foods 2010 2 1 17 22 10.1016/j.jff.2009.12.001

[B16] Higley C Higley A Reference Guide for Essential Oils 2001 London UK: Abundant Health 64

[B17] Adams R Identification of essential oil components by gas chromatography and mass spectroscopy 1995 Carol Stream, Illinois: Allured Publ. Corp Abe S

[B18] Adams RP Quadrupole mass spectra of compounds listed in order of their retention time on DB-5. Identification of Essential Oil Components by Gas Chromatography/Quadrupole Mass Spectroscopy 2001 Illinois: Allured 456

[B19] Shigeru A Naho M Kazumi H Shigeharu I Haruyuki O Hideyou Y Suppression of neutrophil recruitment in mice by geranium essential oil Mediators Inflamm 2004 13 1 21 24 10.1080/09629350410001664798 15203560 PMC1781532

[B20] WHO (World Health Organization) Laboratory Manual for the Examination of Human Semen and Sperm-cervical Mucus Interaction 1999 4 Cambridge: Cambridge University Press

[B21] Kvist U Björndahl L Manual on basic semen analysis. ESHRE Mono-graphs 2 2002 Oxford: Oxford University Press

[B22] Lars B, Trevor GC WHO laboratory manual for examination of the human semen and sperm-cervical mucus interaction 2010 5 Geneva: World Health Organization 5: 7–129

[B23] Wyrobek AJ Bruce WR Chemical induction of sperm abnormalities in mice Proc Nat Acad Sci USA 1975 72 11 4425 4429 10.1073/pnas.72.11.4425 1060122 PMC388734

[B24] Draper HH Hadley M Malondialdehyde determination as index of lipid peroxidation Methods Enzymol 1990 86 421 431 2233309 10.1016/0076-6879(90)86135-i

[B25] Reznick AZ Packer L Methods Enzymol 1994 New York: Academic 357 10.1016/s0076-6879(94)33041-7 8015470

[B26] Aebi H Catalase in vitro Methods Enzymol 1984 105 121 126 6727660 10.1016/s0076-6879(84)05016-3

[B27] Beauchamp C Fridovich I Superoxide dismutase: improved assays and an assay applicable to acrylamide gels Anal Biochem 1971 44 276 287 10.1016/0003-2697(71)90370-8 4943714

[B28] Ellman GL Tissue sulfhydryl groups Arch Biochem Biophys 1959 82 70 77 10.1016/0003-9861(59)90090-6 13650640

[B29] Jollow DJ Mitchell JR Zampaglione N Gillette JR Bromobenzene-induced liver necrosis. Protective role of glutathione and evidence for 3,4-bromobenzene oxide as the hepatotoxic metabolite Pharmacology 1974 11 151 169 10.1159/000136485 4831804

[B30] Jacques-Silva MC Nogueira CW Broch LC Flores EM Ro-cha JBT Diphenyl diselenide and ascorbic changes deposition of selenium and ascorbic in liver and brain of mice Pharmacol Toxicol 2001 88 119 125 10.1034/j.1600-0773.2001.d01-92.x 11245406

[B31] Lowry OH Rosebrough NJ Farr AL Randall RJ Protein measurement with Folin phenol reagent J Biol Chemi 1951 193 265 270 14907713

[B32] Rezvanfar MA Sadrkhanlou RA Ahmadi A Shojaei-Sadee H Rezvanfar MA Mohammadirad A Salehnia A Abdollahi M Protection of cyclophosphamide-induced toxicity in reproductive tract histology, sperm characteristics, and DNA damage by an herbal source; evidence for role of free-radical toxic stress Hum Exp Toxicol 2008 27 901 10.1177/0960327108102046 19273545

[B33] Virendra S Rana Jitendra P Juyal M Amparo B Chemical constituents of essential oil of Pelargonium graveolens leaves The international journal of aromatherapy 2002 12 4 216 218 10.1016/S0962-4562(03)00003-1

[B34] Ben Hsouna A Hamdi N Phytochemical composition and antimicrobial activities of the essential oils and organic extracts from pelargonium graveolens growing in Tunisia Lipids Health Dis 2012 11 167 10.1186/1476-511X-11-167 23216669 PMC3539951

[B35] Boukhris M Monique SJ Simmonds Sayadi S Bouaziz M Chemical composition and biological activities of polar extracts and essential oil of rose-scented geranium, Pelargonium graveolens Phytother Res 2012 Published online in Wiley Online Library (wileyonlinelibrary.com) 10.1002/ptr.4853 23027699

[B36] Al-Sarar AS Abobakr Y Bayoumi AE Hussein HI Al-Ghothemi M Reproductive toxicity and histopathological changes induced by lambda-cyhalothrin in male mice Environ Toxicol 2012 10.1002/tox.21802 22865375

[B37] Cummins JM Jequier AM Kan R Molecular biology of human male infertility: links with aging, mitochondrial genetics, and oxidative stress Mol Reprod Dev 1994 37 362 10.1002/mrd.1080370314 8185940

[B38] Chance B Sies H Boveris A Hydroperoxide metabolism in mammalian organs Physiol Rev 1979 59 605 10.1152/physrev.1979.59.3.527 37532

[B39] Samah S Oda Zeynab Kh E-M Protective effect of vitamin E and selenium combination on deltamethrin-induced reproductive toxicity in male rats Exp Toxicol Pathol 2012 64 813 819 10.1016/j.etp.2011.03.001 21478004

[B40] Yu F Wang Z Ju B Wang Y Wang J Bai D Apoptotic effect of organophosphorus insecticide chlorpyrifos on mouse retina in vivo via oxidative stress and protection of combination of Vitamin C and E Exp Toxicol Pathol 2008 59 415 423 10.1016/j.etp.2007.11.007 18222074

[B41] Nagat A Kawther E-G Fatma M Abdel Khalek E-S Protective effect of vitamin C against chlorpyrifos oxidative stress in male mice Pesticide Biochemistry and Physiology 2010 97 7 12 10.1016/j.pestbp.2009.11.007

[B42] Wang XZ Liu SS Sun Y Wu JY Zhou YL Zhang JH Beta-cypermethrin impairs reproductive function in male mice by inducing oxidative stress Theriogenology 2009 72 599 611 10.1016/j.theriogenology.2009.04.016 19500828

[B43] Cathcart RF Vitamin C: the nontoxic, nonrate-limited, antioxidant free radical scavenger Med Hypothesis 1985 18 61 77 10.1016/0306-9877(85)90121-5 4069036

[B44] Naho M Toshio T Hiroko I Tatsuya H Shigeharu I Hideyo Y Shigeru A Protective activity of geranium oil and its component, geraniol, in combination with vaginal washing against vaginal candidiasis in mice Biol Pharm Bull 2008 31 8 1501 1506 10.1248/bpb.31.1501 18670079

[B45] Keskes-Ammar L Feki-Chakroun N Rebai T Sperm oxidative stress and the effect of an oral Vitamin E and selenium supplement on semen quality in infertile men Arch Androl 2003 49 83 94 12623744 10.1080/01485010390129269

[B46] Sanja Cavar Milka Mak Simovi Antioxidant activity of essential oil and aqueous extract of Pelargonium graveolens L’Her Food Control 2012 23 263 e 267

[B47] Haruyuki O Hideyo Y Suppression of neutrophil recruitment in mice by geranium essential oil Mediators Inflamm 2004 13 1 21 24 10.1080/09629350410001664798 15203560 PMC1781532

[B48] Abe S Maruyama N Hayama K Inouye S Oshima H Yamaguchi H Suppression of neutrophil recruitment in mice by geranium essential oil Mediat Inflamm 2004 13 21 24 10.1080/09629350410001664798 PMC1781532 15203560

[B49] Maruyama N Ishibashi H Hu W Suppression of carrageenan and collagen II-induced inflammation in mice by Geranium oil Mediat Inflamm 2006 53 625 637 10.1155/MI/2006/62537 PMC1592600 16951493

[B50] Boukhris M Bouaziz M Feki I Jemai H El Feki A Sayadi S Hypoglycemic and antioxidant effects of leaf essential oil of Pelargonium graveolens L’Hér. in alloxan induced diabetic rats Lipids Health Dis 2012 11 81 10.1186/1476-511X-11-81 22734822 PMC3439344

